# Construction and analysis of protein–protein interaction networks

**DOI:** 10.1186/1759-4499-2-2

**Published:** 2010-02-15

**Authors:** Karthik Raman

**Affiliations:** 1 Department of Biochemistry, University of Zürich, Winterthurerstrasse 190, 8057 Zürich, Switzerland; 2 Swiss Institute of Bioinformatics, Quartier Sorge, Batiment Genopode, 1015 Lausanne, Switzerland

## Abstract

Protein–protein interactions form the basis for a vast majority of cellular events, including signal transduction and transcriptional regulation. It is now understood that the study of interactions between cellular macromolecules is fundamental to the understanding of biological systems. Interactions between proteins have been studied through a number of high-throughput experiments and have also been predicted through an array of computational methods that leverage the vast amount of sequence data generated in the last decade. In this review, I discuss some of the important computational methods for the prediction of functional linkages between proteins. I then give a brief overview of some of the databases and tools that are useful for a study of protein–protein interactions. I also present an introduction to network theory, followed by a discussion of the parameters commonly used in analysing networks, important network topologies, as well as methods to identify important network components, based on perturbations.

## Introduction

Proteins are the main catalysts, structural elements, signalling messengers and molecular machines of biological tissues [1]. Protein–protein interactions (PPIs) are extremely important in orchestrating the events in a cell. They form the basis for several signal transduction pathways in a cell, as well as various transcriptional regulatory networks. The availability of complete and annotated genome sequences of several organisms has led to a paradigm shift from the study of individual proteins in an organism to large-scale proteome-wide studies of proteins, which interact in a beautifully concerted network of metabolic, signalling and regulatory pathways in a cell. In general, the behaviour of a system is quite different from merely the sum of the interactions of its various parts. As Anderson put it as early as 1972, in his classic paper by the same title, "*More is different*" [2] — it is not possible to reliably predict the behaviour of a complex system, despite a good knowledge of the fundamental laws governing the individual components. Comparative genomics at a primary sequence level has also indicated that species differences are due more to the difference in the interactions between the component proteins, rather than the individual genes themselves [3]. Consequently, several efforts have been made to identify these interactions, in an attempt to understand biological systems better [4-12]. The need to understand protein structure and function has been a critical driving force for biological research in the recent decades. With the advent of high-throughput experiments to identify PPIs, more knowledge on protein function has been obtained, together with the development of several methods to predict and study the interactions between proteins.

A wide variety of methods have been used to identify protein–protein associations; these associations may range from direct physical interactions inferred from experimental methods to functional linkages predicted on the basis of computational analyses. In the past, experimental methods based on microarrays and yeast two-hybrid, as well as computational methods based on protein sequences and structures have been developed and widely used. Given the difficulties in experimentally identifying PPIs, a wide range of computational methods have been used to identify protein–protein functional linkages and interactions. These methods range from identifying a single pair of interacting proteins at one end, to the identification and analysis of a large network of thousands of proteins, the latter as large as that of an entire proteome of a given cell.

## Computational methods for prediction of protein–protein functional linkages and interactions

### Methods based on genomic context

#### Domain fusion

**Figure 1:**
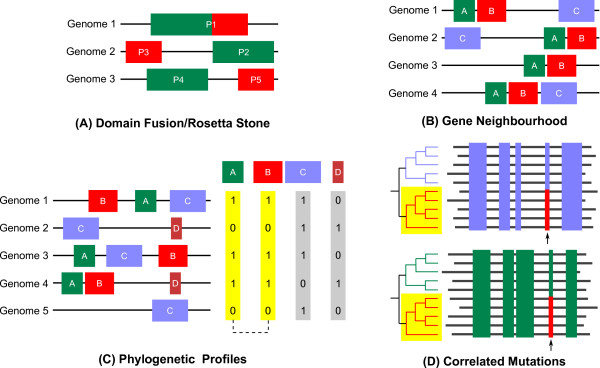
**Prediction of functional linkages between proteins, based on different methods**. **(A) Method of domain fusion**. The figure shows proteins predicted to interact by the Rosetta stone method (domain fusion). Each protein is shown schematically with boxes representing domains. Proteins P2 and P3 in Genomes 2 and 3 are predicted to interact because their homologues are fused in the first genome. **(B) Gene neighbourhood**. The figure shows four hypothetical genomes, containing one or more of the genes A, B and C. Since the genes A and B are co-localised in multiple genomes (1–4), they are likely to be functionally linked with one another. **(C) Phylogenetic profiles**. The figure shows five hypothetical genomes, each containing one or more of the proteins A, B, C and D. The presence or absence of each protein is indicated by 1 or 0, respectively, in the phylogenetic profiles given on the right. Identical profiles are highlighted — proteins A and B are functionally linked (dotted line), whereas proteins C and D, which have different phylogenetic profiles (shown in grey) are not likely to be functionally linked. **(D) Correlated mutations**. The alignments of two protein families are shown; conserved residues in either alignment are shown in the same colour (blue and green). Correlated mutations in either alignment (coloured red) are indicated by arrow marks. Common sub-trees of the phylogenetic trees are highlighted in yellow. The presence of correlated mutations in each family suggests that the corresponding sites may be involved in mediating interactions between the proteins from each family.

The domain fusion or Rosetta Stone method was proposed by Eisenberg and co-workers [13]. The method is based on the hypothesis that if domains ***A ***and ***B ***exist fused in a single polypeptide ***AB ***in another organism, then ***A ***and ***B ***are functionally linked. Fig. 1A shows an example to illustrate this point. The premise is that since the affinity between proteins ***A ***and ***B ***is greatly enhanced when ***A ***is fused to ***B***, some interacting pairs of proteins may have evolved from proteins that included the interacting domains ***A ***and ***B ***on the same polypeptide. Veitia [14] has proposed a kinetic background to the idea of gene fusion, suggesting the inclusion of eukaryotic sequences to increase the robustness of Rosetta Stone predictions. The argument basically involves the fact that eukaryotes, with a larger volume, cannot afford to accommodate separate proteins ***A ***and ***B***, as the required concentrations of ***A ***and ***B ***would be prohibitively high, to achieve the same equilibrium concentration of ***AB***. One limitation of this method is its low coverage; it has the least coverage among the methods based on genomic context [15].

#### Conserved neighbourhood

If the genes that encode two proteins are neighbours on the chromosome in several genomes, the corresponding proteins are likely to be functionally linked [16]. This method is particularly useful in case of prokaryotes, where operons commonly exist, or in organisms where operon-like clusters are observed. Fig. 1B shows an example to illustrate this method. This method has been reported to identify high-quality functional relationships [17]. However, the method suffers from low coverage, due to the dual requirement of identifying orthologues in another genome and then finding those orthologues that are adjacent on the chromosome [17]. Nevertheless, this coverage is still higher than that of the Rosetta Stone method [15]. Bork and co-workers have proposed another approach that exploits the conservation of divergently (bi-directionally) transcribed gene pairs [18]. The method is complementary to the existing gene neighbourhood method, which focuses on operons, where the genes are transcribed in a common orientation (co-directionally). They report the application of this method, to successfully associate self-regulatory transcription factors to their respective operons, enhancing functional annotations [18].

#### Phylogenetic profiles

Identification of functional linkages between proteins using phylogenetic profiles is based on the idea that functionally linked proteins would co-occur in genomes. The phylogenetic profile of a protein can be represented as a 'bit string', encoding the presence or absence of the protein in each of the genomes considered (see Fig. 1C). Proteins having matching or similar phylogenetic profiles tend to be strongly functionally linked [19]. In a study reported in 1999 [19], when only 17 fully sequenced genomes were considered for analysis, the function of a number of proteins in *Escherichia coli *could be assigned correctly, by examining the similarity of their phylogenetic profiles. Fig. 1C illustrates an example, showing how two proteins ***A ***and ***B ***are likely to be functionally linked, owing to the similarity of their phylogenetic profiles across five genomes. This method is in a sense the computational equivalent of the experimental genetic approach of mapping a mutant gene's phenotype to the gene. Genes with similar phylogenetic profiles essentially produce similar phenotypes, much similar to a standard genetic mapping [17]. Bork and co-workers [20] have used anti-correlated occurrences of genes (complementary phylogenetic patterns, as against co-occurrence) across genomes to identify several analogous enzyme displacements (functionally equivalent genes) in thiamine biosynthesis.

The online service Protein Link EXplorer (PLEX; http://bioinformatics.icmb.utexas.edu/plex/) [21] allows for the construction of phylogenetic profiles for any given sequence, which can be compared to profiles of all other proteins from 89 fully sequenced genomes that are stored in the PLEX database. PLEX can also accept sophisticated phylogenetic profile inputs and comparison parameters, including individual organism or group-based profiles. Gene neighbours and Rosetta stone links of all proteins that match the query profile can also be investigated.

### Methods based on co-evolution

Co-evolution can be defined as the joint evolution of ecologically interacting species [22] and it implies the evolution of a species in response to selection imposed by another. Co-evolution thus requires the existence of mutual selective pressure on two or more species [23]. Computational methods to predict PPIs through the characteristics of co-evolution have been developed by extrapolating concepts developed for the study of species co-evolution to the molecular level [23,24]. An *in silico *Two-hybrid (*i2h*) method has been proposed, based on the study of correlated mutations in multiple sequence alignments [25,26]. The premise is that co-adaptation of interacting proteins can be detected by the presence of a distinctive number of compensatory mutations in corresponding proteins of different species. An interaction index, defined based on the distribution of correlation values is calculated. Correlated mutations can also been used to identify specific residues involved at the interaction sites [26]. Fig. 1D illustrates how correlated mutations can be used to identify functional linkages between proteins.

Protein interactions have also been predicted on the basis of the comparison of evolutionary histories, or phylogenetic trees, under the premise that interacting proteins are subject to similar evolutionary pressures resulting in similar topologies for the corresponding trees [27-29]. A more recent method [30] uses the complete network of phylogenetic tree similarities between all protein pairs in the genome to reassess pairwise similarity between the phylogenetic trees of any two proteins, thereby accounting for the co-evolutionary context of the proteins more effectively.

### Other methods

Although homology-based methods are often quite useful for inferring PPIs, there are occasions where homology-based methods may not be effective. For example, Mika and Rost have illustrated earlier that homology-based inference of physical PPIs are accurate only at high levels of sequence identity [31]. Further, homology-based inference of PPIs work better within species than across species, for low and high levels of sequence similarity [31].

Functional linkages may also be derived by the analysis of correlated mRNA expression levels, or protein co-expression. These techniques do not require any homology information [17], as they rely on the measurement of additional expression data. These techniques can, therefore, find unique relationships among proteins. The premise of all expression clustering methods is that proteins do not work in isolation and are often co-expressed with functionally related proteins. By altering the conditions for performing the experiments, enough variation in gene expression can be observed to identify co-expressing genes. Protein co-expression analysis is preferable since mRNA levels and protein levels have often been found to be poorly correlated.

Gene expression data has also been shown to be useful in understanding the dynamics of PPI networks [32-34]. Lu and collaborators [33] integrated gene expression profiles (from a mice model of asthma) into a network of mouse PPIs derived from the BIND database. They found that highly connected proteins, or hub proteins in the network have less variable gene expression profiles compared to proteins at the network periphery. Mande and collaborators have described the construction of 'conditional networks' by integrating gene expression data under different conditions into protein functional linkage networks [34]. These networks present a picture of the dynamics of the functional linkages between proteins; a comparative analysis of four different conditional networks illustrates important responses in wild-type and mutant *Escherichia coli *cells treated with ultra-violet rays.

Efforts to mine experimental protein–protein association information from literature have also been made. For example, Hogue and co-workers have described an support vector machine (SVM)-based approach to mine the biomedical literature for PPIs [35]. Databases such as the STRING include such computationally mined interactions [36]. Eisenberg and co-workers have described an approach to identify abstracts that discuss PPIs from literature, which may then be manually scanned to identify PPIs [37]. This approach forms the basis for the rapid expansion of the database of interacting proteins (DIP) [37]. Zaki and collaborators have described a method based on pairwise similarity of protein sub-sequences, to predict PPIs [38].

### Experimental methods

Although this review primarily deals with computational methods for predicting PPIs, I here briefly outline some experimental methods for assessing PPIs, for the sake of completeness. There are a number of experimental techniques such as yeast-two hybrid [39], affinity purification/mass spectrometry [4,5,9,11,40] and protein microarrays [41-43], which are reviewed in detail elsewhere [44,45]. These form the basis of several large-scale datasets on PPIs.

In the yeast-two hybrid assay, two fusion proteins are created: the 'bait' (a protein of interest with a DNA-binding domain attached to its N-terminus) and the 'prey' (its potential interaction partner, fused to an activation domain). If the 'bait' and the 'prey' interact, their binding forms a functional transcriptional activator, which in turn activates reporter genes or selectable markers [39]. This assay has been adapted for high-throughput analyses of PPIs [46,47].

Gavin and collaborators have described the purification of complexes of 1739 proteins from *S. cerevisiae *(including the complete set of 1143 human orthologues) using tandem affinity purification coupled to mass spectrometry, illustrating the complexity of connectivity between protein complexes [4]. Mass spectrometry has also been used to construct a large-scale map of human protein interactions [11].

Protein microarrays aid in the detection of *in vitro *binary interactions of various types — protein–protein, protein–lipid or antigen–antibody interactions. Proteins covalently attached to a solid support are screened with fluorescently labelled probes (proteins or lipids), to identify interactions [41]. A high density yeast protein microarray comprising 5800 yeast proteins was developed and used to identify novel calmodulin and phospholipid binding proteins [41].

Although many of these assays can identify PPIs with high confidence, they still have their share of false positives and can suffer from a limited reproducibility. Nevertheless, high-throughput experimental analyses of PPIs are quite important in obtaining the protein interaction map of a cell. Further, combining results from multiple experiments as well as computational methods for predicting functional linkages (as is done in databases such as the STRING) is likely to further improve our understanding of the complex web of interactions within a cell.

## Databases and tools for analysis of PPIs

In this section, I review some of the important databases that house data on PPIs, as well as some useful tools for the visualisation and analysis of PPIs. Protein interaction databases have also been reviewed in [44]. Some of the important databases containing data about PPIs are discussed below. Some more examples of databases useful for researching PPIs are given in Table 1.

**Table 1:** Databases and resources useful for researching PPIs.

Database	URL	Resources	Refs.
BIND	Peer-reviewed bio-molecular interaction database containing published interactions and complexes	http://bind.ca/	[79]
BioGRID	Protein and genetic interactions from major model organism species	http://www.thebiogrid.org/	[80]
COGs	Orthology data and phylogenetic profiles	http://www.ncbi.nlm.nih.gov/COG/	[81,82]
DIP	Experimentally determined interactions between proteins	http://dip.doe-mbi.ucla.edu/	[51]
HPRD	Human protein functions, PPIs, post-translational modifications, enzyme–substrate relationships and disease associations	http://www.hprd.org/	[50,83]
IntAct	Interaction data abstracted from literature or from direct data depositions by expert curators	http://www.ebi.ac.uk/intact/	[84]
iPFAM	Physical interactions between those Pfam domains that have a representative structure in the Protein DataBank (PDB)	http://ipfam.sanger.ac.uk/	[85]
MINT	Experimentally verified PPI mined from the scientific literature by expert curators	http://mint.bio.uniroma2.it/mint/	[86]
Predictome	Experimentally derived and computationally predicted functional linkages	http://visant.bu.edu/	[52]
ProLinks	Protein functional linkages	http://mysql5.mbi.ucla.edu/cgi-bin/functionator/pronav	[87]
SCOPPI	Domain–domain interactions and their interfaces derived from PDB structure files and SCOP domain definitions	http://www.scoppi.org/	[88]
STRING	Protein functional linkages from experimental data and computational predicttions	http://string.embl.de/	[36,48]

### STRING

STRING (Search Tool for the Retrieval of Interacting Genes/Proteins; http://string.embl.de/) [36,48] is a pre-computed database for the exploration and analysis of protein–protein associations. The associations are derived from high-throughput experimental data, mining of databases and literature, analyses of co-expressed genes and also from computational predictions, including those based on genomic context analysis. STRING employs a unique scoring framework based on benchmarks of the different types of associations against a common reference set, to produce a single confidence score per prediction. The graphical user interface is appealing and user-friendly, backed by an excellent visualisation engine. Medusa http://coot.embl.de/medusa/, a general graph visualisation tool, is a front end (interface) to the STRING protein interaction database [49].

### HPRD

Human Protein Reference Database (HPRD; http://www.hprd.org/) [50] integrates information relevant to the function of human proteins in health and disease. The database is almost completely manually curated by biologists who have read and interpreted over 300,000 published articles during the annotation process. Data pertaining to thousands of PPIs, post-translational modifications, enzyme/substrate relationships, disease associations, tissue expression and sub-cellular localisation have been extracted from literature into the database.

### DIP

The DIP (Database of Interacting Proteins; http://dip.doe-mbi.ucla.edu/) database [51] catalogues experimentally derived PPIs. Due to the variety of experiments and their corresponding reliabilities, DIP applies some quality assessment methods to pick out subsets of most reliable interactions. The DIP is generally considered as a valuable benchmark or verify the performance of any new method for prediction of PPIs.

### Predictome

The Predictome [52] database houses links between the proteins of 44 genomes based on the implementation of gene context functional linkage methods, viz. chromosomal proximity, phylogenetic profiling and domain fusion. It also contains information on large-scale experimental screenings of PPI data, from experiments such as yeast two-hybrid, immuno-co-precipitation and correlated expression. The Predictome database is presently accessible through the visual front-end provided by VisANT [53], which is a versatile tool for visualisation and analysis of interaction data. Website http://visant.bu.edu/.

#### Tools for network analysis and visualisation

In this section, I briefly discuss some of the useful software tools available for the analysis and visualisation of biological networks. A comprehensive review of the tools useful for the visualisation of networks has been published elsewhere [54]. Some more examples of tools useful for network visualisation and analysis are given in Table 2.

**Table 2:** Examples of tools useful for the visualisation of networks and PPIs.

Tool	URL	Features	Refs.
BioLayout Express 3D	http://www.biolayout.org/	Facilitates microarray data analysis	[89]
Cytoscape	http://www.cytoscape.org/	Versatile; implements many visualisation algorithms; many plug-ins available	[55]
Large Graph Layout (LGL)	http://sourceforge.net/projects/lgl	Especially useful for dynamic visualisation of large graphs (10^5 ^nodes, 10^6 ^edges); force-directed layout algorithm	[90]
Osprey	http://biodata.mshri.on.ca/osprey/servlet/Index	Provides network filters, connectivity filters, many layouts and facilitates dataset superimposing	[91]
Pajek	http://vlado.fmf.uni-lj.si/pub/networks/pajek/	Especially useful for the analysis of very large networks	[92]
Visant	http://visant.bu.edu/	Especially facilitates analysis of gene ontologies	[53]
Yed	http://www.yworks.com/products/yed/	General purpose graph editor	-

##### Cytoscape

Cytoscape http://www.cytoscape.org/[55] is a software platform for visualising molecular interaction networks and integrating these interactions with gene expression profiles. The tool is best used in conjunction with large databases of gene expression data, protein–protein, protein–DNA, and genetic interactions that are increasingly available for humans and model organisms. Cytoscape supports several algorithms for the layout of networks. Several useful plug-ins are available for Cytoscape, to extend its capabilities. A notable example is the NetworkAnalyzer plug-in [56], which can be used to compute various network parameters.

##### Pajek

Pajek http://pajek.imfm.si/ is a program (only for Windows-based operating systems) for the analysis and visualisation of very large networks; it can even handle networks with > 10^5 ^nodes. Pajek also includes a variety of network layout algorithms, including force-directed layout algorithms such as Fruchterman–Reingold [57]. Pajek is highly versatile and can also be used to study network dynamics.

## Analyses of network structure

The field of network theory has witnessed a number of advances in the past [58-60], many of which are impacting the analyses of biological networks such as PPI networks. In this section, I discuss some of the important network parameters useful in the analysis of networks and understanding their characteristics, important network topologies, as well as some of the measures that can be used to analyse perturbations to networks. Detailed reviews of the application of network theory to biology have been published elsewhere [61,62].

### Network parameters

Network theory provides a quantifiable description of networks; there are several network measures that enable the comparison and characterisation of complex networks:

#### Connectivity (or) Degree

The most elementary characteristic of a node is its degree, *k*, which represents the number of links the node has, to other nodes in the network.

#### Degree distribution

The degree distribution, *P*(*k*), gives the probability that a selected node has exactly *k *links. *P*(*k*) is obtained by counting the number of nodes *N*(*k*) with *k *= 1, 2, ... links and dividing by the number of nodes *N*. The degree distribution allows to distinguish between various network topologies [61].

#### Clustering Coefficient

The clustering coefficient was first defined by Watts and Strogatz [58]. The clustering coefficient, *C*, for a node is a notion of how connected the neighbours of a given node are (*cliquishness*). The average clustering coefficient for all nodes in a network is taken to be the network clustering coefficient. In an undirected graph, if a vertex *v*_*i *_has *k*_*i *_neighbours, *k*_*i*_(*k*_*i *_- 1)*/*2 edges could exist among the vertices within the neighbourhood (*N*_*i*_). The clustering coefficient for an undirected graph **G**(**V**, **E**) (where **V **represents the set of vertices in the graph **G **and **E **represents the set of edges) can then be defined as(1)

The average clustering coefficient characterises the overall tendency of nodes to form clusters or groups. *C*(*k*) is defined as the average clustering coefficient for all nodes with *k *links.

#### Characteristic Path Length

The characteristic path length, *L*, is defined as the number of edges in the shortest path between two vertices, averaged over all pairs of vertices. It measures the typical separation between two vertices in the network [58]. Intuitively, it represents the network's overall navigability [61].

#### Network Diameter

The network diameter *d *is the greatest distance (shortest path, or geodesic path) between any two nodes in a network [63]. It can also be viewed as the length of the 'longest' *shortest path *in the network.(2)

where *d*_**G**_(*u, v*) is the shortest path between *u *and *v *in **G**. A few authors have also used this term to denote the average geodesic distance in a network (which translates to the characteristic path length), although strictly the two measures are distinct.

#### Betweenness

Betweenness is a centrality measure of a vertex within a graph [64]. For a graph **G**(**V**, **E**), with *n *vertices, the betweenness *C*_*B*_(*v*) of a vertex *v *is defined as(3)

where *σ*_*st *_is the number of shortest paths from *s *to *t*, and *σ*_*st*_(*v*) is the number of shortest paths from *s *to *t *that pass through a vertex *v*. A similar definition for 'edge betweenness' was given by Girvan and Newman [65]. Nodes with a higher betweenness lie on a larger number of shortest paths in a network.

### Network topologies

The understanding of the topology or the architectural principles of a biological network can directly give an insight into various network characteristics. There are several known topologies of networks, characterised by their distinctive network parameters. The following are some network models that are relevant to the understanding of biological networks.

#### Random networks

The Erdös–Rényi model of a random network starts with *N *nodes and connects each pair of nodes with a probability *p*, which creates a graph with approximately *pN*(*N *- 1)*/*2 randomly placed links. The node degrees follow a Poisson distribution indicating that most nodes have approximately the same number of links. The characteristic path length is proportional to the logarithm of the network size *L *~ log *N*. *C*(*k*) is independent of *k *[61].

#### Small-world networks

Small-world networks are characterised by two properties: (i) individual nodes have few neighbours, but (ii) most nodes can be reached from one another through few steps, often referred to as 'six degrees of separation' [66]. Small-world networks have been generated by re-wiring regular ring-lattice-like networks [58]. A regular ring-lattice resembles a (circular) string of beads, where each node (bead) is linked to one node on either side, and is also additionally connected to the immediate neighbour of those nodes. Thus, each node is linked to four nodes nearest to it on the 'string'. The ring-lattice is rewired as follows: the original links in the lattice are replaced by random ones with a probability 0 ≤ *ϕ *≤ 1, introducing varying amounts of disorder, which takes the network from complete regularity to complete disorder (randomness). The re-wiring process allows the small-world model to interpolate between a regular lattice and a (more or less) random graph. When *ϕ *= 0, there is no re-wiring and the regular lattice remains unchanged. The clustering coefficient for this lattice tends to 0.75 for large *k*. The regular lattice, however, does not show the small-world effect. Mean geodesic distances between vertices tend to *L/*4*k *for large *L*. When *ϕ *= 1, every edge is re-wired to a new random location and the graph is almost a random graph, with typical geodesic distances on the order of log *L/ *log *k*, but very low *C *≃ 2*k/L *[67]. As Watts and Strogatz showed by numerical simulation, however, there exists a sizeable region in between these two extremes of *ϕ*, for which the model generates a network that has both low path lengths and high clustering. Small-world networks have a characteristic path length of the same order as random networks (*L *≳ log *N*), but have a clustering coefficient much higher than that of random networks (*C *≫ *C*_random_). The small-world topology has been observed in networks such as film actor networks, power grids and the neural network of the nematode *Caenorhabditis elegans *[58].

#### Scale-free Networks

Scale-free networks are characterised by a power-law degree distribution; the probability that a node has *k *links is given by *P*(*k*) ~ *k*^-*γ*^, where *γ *is the degree exponent [59]. The value of *γ *determines many properties of the system. For smaller values of *γ*, the role of the 'hubs', or highly connected nodes, in the network becomes more important. For *γ *> 3, hubs are not relevant, while for 2 <*γ *< 3, there is a hierarchy of hubs, with the most connected hub being in contact with a small fraction of all nodes. Scale-free networks have a high degree of robustness against random node failures, although they are sensitive to the failure of hubs. The probability that a node is highly connected is statistically more significant than in a random graph. The properties of a scale-free network are often determined by a relatively small number of highly connected hubs. The Barabási–Albert scale-free network model [59] involves the construction of a network through an iterative procedure. Beginning with a network having *m*_0 _nodes, in each subsequent iteration, a single node is added to the network, with *m *≤ *m*_0 _links to existing nodes. The probability with which this node connects to the existing nodes of the network is directly proportional to the connectivity of the existing nodes ('*rich get richer*' phenomenon). The probability *p*_*i *_with which the new node connects to an existing node *i*, is given as

where *k*_*i *_is the degree of node *i *and the denominator represents the sum of the degrees of all nodes in the network (**G**). After *n *iterations, the model leads to a network with *m*_0 _+ *n *nodes and *mn *edges. The network generated by this model has a power-law degree distribution characterised by *γ *= 3. Scale-free networks with 2 <*γ *< 3, a range commonly observed in many biological networks, are ultra-small, with a characteristic path length *L *~ log log *N*, significantly smaller than that of random networks (log *N*) [61].

### Analysis of network perturbations

Networks can be perturbed through the removal of nodes and edges. A typical analysis would be to probe the effect of disrupting a node and its corresponding edges. Networks of different topologies vary in their resilience to various types of perturbations. A number of studies have been carried out to analyse the response of networks to the deletion of their nodes and edges. A review of how nodes in a network can be prioritised based on network analysis has been presented elsewhere [68].

Barabási and co-workers have analysed the response of scale-free and random networks to various types of 'attacks' [69]. In particular, they have analysed the networks representing the topologies of the Internet and the World-Wide Web. The common observation is that scale-free networks are quite insensitive to random node removals; they are highly robust in the face of random node failures and the characteristic path length was found to be almost unaffected. This is intuitively reasonable, since most of the vertices in these networks have low degree and therefore lie on few paths between others; thus their removal rarely affects communications substantially. On the other hand, directed attacks targeting the highly connected hubs led to a rapid disruption of the communication through the network. The characteristic path length was found to increase very sharply with the fraction of hubs removed and typically only a small fraction of the hubs needed to be 'knocked out' before essentially all communication through the network was destroyed [67,69].

Jeong and co-workers have analysed the effect of node deletions on *S. cerevisiae *PPI network [70]. They report that although proteins with five or fewer links constituted about 93% of the total number of proteins, only about 21% of them were essential. On the other hand, only 0.7% of the proteins had more than 15 links, but single deletion of 62% of these proved lethal. This implies that highly connected proteins with a central role in the architecture of the network are three times more likely to be essential than proteins with only a small number of links to other proteins.

Another comprehensive analysis of vulnerability of complex networks to various types of attacks has been discussed in [71]. In addition to node deletions studied earlier [69], they have also studied the effects of edge removals. Further, for each case of attacks on vertices and edges, four different attacking strategies were employed: removals by the descending order of the degree and the betweenness centrality, calculated for either the initial network or the modified network during the iterative removal procedure. They report that the removals based on the re-calculated degrees and betweenness centralities are often more harmful than the attack strategies based on the initial network's parameters, underlining the importance of the changes in network structure following the removal of important edges or nodes.

Wingender and co-workers have proposed a measure, known as *pairwise disconnectivity index *[72], which quantifies how crucial a node or an edge (or a group of nodes/edges) is, for sustaining the communication between connected pairs of vertices in a directed network. This is one metric that explicitly considers paths between the various nodes in a network; it is thus quite useful in analysing how node deletions in a network can disrupt the flow of information.

We have earlier reported an analysis of the number of disrupted shortest paths in the network, to identify nodes that may be critical to a network [73]. Network analysis has also been used for identifying pathways to drug resistance [74]. Ge and collaborators have developed an 'information flow analysis', to identify proteins central for information transmission in interactome networks of *S. cerevisiae *and *C. elegans *[75]; the proteins so identified were also likely to be essential for survival. The method employs confidence scores for PPIs and also considers multiple paths in a network while evaluating the importance of each protein [75]. The analysis of node deletions from PPI networks has been used for the identification of potential drug targets [73,76].

## Conclusions

PPI networks provide a simplified overview of the web of interactions that take place inside a cell. The vast amounts of sequence data that have been generated have been leveraged to make better predictions of interactions and functional associations between proteins, as well as individual protein functions. By integrating experimental methods for determining PPIs and computational methods for prediction, a lot of useful data on PPIs have been generated, including a number of high-quality databases.

Although the analyses of PPI networks has produced several useful results, often improving our understanding of the underlying biology, they are not without flaws. One of the key flaws of the existing methods to delineate such large-scale protein interaction networks is the limited reproducibility of such experiments; further, it is suspected that what is examined is only a small fraction of the entire proteome [77]. However, most databases do combine multiple methods for predicting interactions, as well as results from multiple high-throughput experiments, mitigating this problem to a certain extent. Further, these networks often paint a static picture of the overwhelmingly complex dynamic interactions that take place in a cell. An improved model of these interactions must consider both the dynamics (temporal changes in the interactions) as well as the strengths of each of the interactions. The global overview presented by such interaction maps is no doubt useful, but the finer details of the interactions may be significantly important for our ability to make testable predictions about biological systems [78].

Nevertheless, protein interaction maps have many practical applications and hold the key to understanding complex biological systems. With a large amount of high-throughput data being generated at various levels, computational analyses of these data, to identify associations and interactions between various proteins, form a fundamental step in our quest to understand the organisation of complex biological systems. As Dennis Bray put it rather eloquently [78], "*We have a new continent to explore and will need maps at every scale to find our way*".

## Competing interests

The author declares that he has no competing interests.

## Authors' contributions

KR wrote, read and approved the final manuscript.
